# Supplementation of Rumen-Protected Glucose Increased the Risk of Disturbance of Hepatic Metabolism in Early Postpartum Holstein Cows

**DOI:** 10.3390/antiox11030469

**Published:** 2022-02-26

**Authors:** ZhiYuan Ma, LuoYun Fang, Emilio Ungerfeld, XiaoPeng Li, ChuanShe Zhou, ZhiLiang Tan, LinShu Jiang, XueFeng Han

**Affiliations:** 1 CAS Key Laboratory for Agro-Ecological Processes in Subtropical Region, National Engineering Laboratory for Pollution Control and Waste Utilization in Livestock and Poultry Production, South-Central Experimental Station of Animal Nutrition and Feed Science in Ministry of Agriculture, Institute of Subtropical Agriculture, The Chinese Academy of Sciences, Changsha 410125, China; mzy@lzu.edu.cn (Z.M.); lixiaopeng123lover@163.com (X.L.); zcs@isa.ac.cn (C.Z.); zltan@isa.ac.cn (Z.T.); 2 College of Pastoral Agriculture Science and Technology, Lanzhou University, Lanzhou 730000, China; 3 Beijing Key Laboratory for Dairy Cow Nutrition, Beijing University of Agriculture, Beijing 102206, China; fangly@bac.edu.cn; 4 Centro Regional de Investigación Carillanca, Instituto de Investigaciones Agropecuarias INIA, Vilcún 4880000, Chile; emilio.ungerfeld@inia.cl

**Keywords:** rumen-protected glucose, liver, oxidative stress, proteomics

## Abstract

The dual stress of reduced feed intake and increased milk yield in dairy cows early postpartum results in a negative energy balance. Rumen-protected glucose (RPG) has been reported to replenish energy, increase milk yield, and improve gut health. However, early postpartum cows often develop an insulin resistance, implying that RPG may not be well utilized and increased milk production may increase the liver’s fat oxidization burden. This study aimed to investigate the effects of RPG on the hepatic oxidative/antioxidative status and protein profile. Starting 7 d before expected calving, six pairs of cows were supplemented with rumen-protected glucose (RPG, *n* = 6) or with an equal amount of rumen-protecting coating fat (CON, *n* = 6). Liver samples were obtained from 10 cows 14 d after calving (d 14). Concentration of malondialdehyde and activity of glutathione peroxidase were increased and the activities of catalase and superoxide dismutase tended to increase in the livers of the RPG cows compared to the CON cows. The revised quantitative insulin sensitivity check index (RQUICKI) was decreased by RPG, but triacylglycerol concentration in liver was increased by RPG supplementation. The overall profiles of hepatic proteins were similar between CON and RPG. A partial least square regression was conducted to identify the proteins associated with liver lipidosis, oxidative stress, and antioxidative capacity. The top twenty proteins, according to their variable importance value, were selected for metabolic pathway enrichment analysis. Eighteen enriched KEGG pathways were identified, including metabolism, the citrate cycle, propanoate metabolism, the peroxisome, and type II diabetes mellitus. Our study showed that RPG supplementation reduced insulin sensitivity but increased the liver triglyceride concentration and the oxidative stress in early postpartum cows. Liver proteins related to lipidosis, oxidative stress, and antioxidative capacity, were positively associated with the glutamine metabolism, citric acid cycle, peroxisome, and type II diabetes pathways, which may indicate an increased risk of liver metabolic disorders caused by RPG supplementation in early postpartum cows.

## 1. Introduction

Early postpartum dairy cows experience a negative energy balance under the dual stress of insufficient energy intake due to anorexia and the increased energy demand for milk production [1,2]. The negative energy balance in the early postpartum dairy cow is associated with metabolic disorders such as ketosis and hepatic lipidosis [3,4,5].

Considerable research has been conducted seeking means to alleviate the negative energy balance: dietary supplementation with cereals [6], linoleic acid [7], rumen-protected methionine [8], and glucose [9]. In our previous studies, fat-coated, rumen-protected glucose (RPG) was supplemented to dairy cows during the transition period, resulting in an improved milk production [1] and gut health [10]. However, increased non-esterified fatty acids (NEFA) in plasma due to RPG supplementation, an indication of excessive lipolysis, was also observed [1]. Excessive lipolysis is a result of insufficient energy intake and mobilization of the body’s energy reserves [11]. Fat mobilization from adipose tissue can exceed the oxidative capacity of the liver, resulting in metabolic disorders such as fatty liver disease, an increase in reactive oxygen species (ROS), a reduction of paraoxonase activity, and the onset of oxidative stress [12]. Rumen-protected glucose supplementation has been hypothesized to ameliorate the energy deficit but was instead shown to increase the negative energy balance of periparturient dairy cows [1]. We speculate that supplemented glucose in the form of RPG may stimulate the mammary gland to produce more milk, leading to a greater energy demand.

The liver is a hub organ in energy metabolism, with its activity being tightly controlled by hormones such as insulin [13]. Mammals in early postpartum tend to develop liver insulin resistance [14], which may be one of the mechanisms through which RPG failed to alleviate the negative energy balance of periparturient cows in a study by Li et al. (2019). It is possible that the supply of exogenous energy can either cause or aggravate liver metabolic disorders in transition cows, but that remains to be studied.

Recent advances in proteomic technologies have allowed researchers to characterize the proteomic profiles of the livers of dairy cows [15,16]. Tandem mass tags (TMT) technology, combined with UPLC-MS/MS, is a powerful technique for protein screening and is particularly well suited for small sample sizes [17]. In this study, TMT-based quantitative proteomic analyses were performed in the livers of early lactation Holstein cows to evaluate the effects of RPG supplementation. This study aimed to gain a better understanding of the effects of RPG on the hepatic oxidative/antioxidative status and protein profile.

## 2. Materials and Methods

### 2.1. Animals and Experimental Design

The animal trial was described in one of our previously published papers [1]. Out of a total of 22 cows with an expected calving date between May and July 2018, twelve 4–5 yr old Holstein cows (515 ± 42 kg body mass, 16.1 ± 3.7 kg milk/d, 2.5 ± 0.52 parity; mean ± SD) were selected for this animal trial. The twelve cows were partitioned into six pairs based on their milk production in the previous lactation cycle. One cow per pair was randomly assigned to either the control group (CON, *n* = 6) or to the rumen-protected glucose group (RPG, *n* = 6). The CON cows were fed the basal diet (Supplemental Table S1) plus 90 g/d of coating fat, and the RPG supplemented cows were fed the basal diet supplemented with 200 g of RPG, composed of 90 g glucose as the core with 90 g of coating fat and 20 g of water. One of the RPG supplemented cows developed acute cholecystitis unrelated to her treatment, so she was excluded from the trial along with her paired CON cow. All cows were individually fed the basal diet as a total mixed ration (TMR) twice daily at 07:30 and 14:30 h, allowing for a 10% refusal rate. Either the coating fat or RPG was provided to the corresponding cows from 7 d before calving to 14 d postpartum. The daily amount of coating fat and RPG were split into the two daily meals. A portion of the TMR mixed with the coating fat or RPG was fed first. The rest of the TMR without the coating fat or RPG was offered after the first portion of TMR containing either the coating fat or RPG was consumed. Water was freely available at all times.

### 2.2. Insulin Resistance Estimate

At 14 d postpartum, jugular venous blood was sampled before the morning feeding and after a 10 h overnight fasting. Concentrations of glucose, insulin, and NEFA in blood were measured as in our previous report [1]. A revised quantitative insulin sensitivity check index (RQUICKI) was calculated based on the reciprocal of the sum of the logarithm-transformed glucose, insulin, and NEFA concentrations [18]:

$$\mathrm{RQUICKI}=\frac{1}{\log10\left(G_{b}\right)+\log10\left(I_{b}\right)+\log10\left(NEFA_{b}\right)}$$

where *G_b_* (mg/dL), *I_b_* (μU/mL), and *NEFA_b_* (mM) are 10 h fasting concentrations of glucose, insulin, and NEFA in blood, respectively.

### 2.3. Liver Samples Collection

Five cows from each treatment were humanely euthanized with sedative xylazole at 14 d postpartum, and about 30 g of liver tissue was obtained from the middle of the right lobe. Bloodstains on the surface of the liver samples were rinsed with cold sterile saline. Samples were then divided into small pieces of about 2 g each and individually wrapped in sterile tin foil before being placed into sampling bags (Whirl-Pak™, Madison, WI, USA), which were quickly immersed in liquid nitrogen. The frozen liver samples were then transferred to a −80 °C refrigerator for long-term storage until analysis.

### 2.4. Liver Antioxidant Capacity and Triglyceride Measurement

Samples of liver tissue were ground with ice-cold saline (1/10, *v*/*v*). The resulting homogenates were then centrifuged at 1200× *g* at 4 °C for 10 min. The supernatants were then transferred to new Eppendorf tubes, and the protein concentration was determined through a Bradford assay. All oxidative stress and antioxidant indicators in liver samples were measured using commercially available kits provided by Beyotime (Shanghai, China). Malondialdehyde (MDA) concentration was determined by color reaction with thiobarbituric acid. Catalase (CAT) activity was determined by measuring the decrease in H_2_O_2_ concentration observed following incubation of the sample with an H_2_O_2_ standard solution. Total antioxidant capacity was determined using the ABTS method [19]. Total superoxide dismutase (SOD) activity was determined using the WST-8 method [20]. Activity of total glutathione peroxidase (GPx) was determined using the NADPH method [21]. Determination of liver triglyceride concentration was conducted according to the GPO-PAP method [22], using a kit provided by the Nanjing Jiancheng Biological Engineering Institute (Nanjing, China).

### 2.5. TMT-Based Quantitative Proteomics Analysis

#### 2.5.1. Protein Pre-Treatment and TMT Labeling

Frozen liver samples were ground to powder in liquid nitrogen. Twenty milligrams of frozen liver powder were mixed with a 1.5 mL cold mix of tri-n-butyl phosphate/acetone/methanol (1:12:1, *v*/*v*/*v*) and left for 90 min at 4 °C to remove the lipids. Samples were then centrifuged at 10,000× *g* and 4 °C for 20 min. Pellets were air-dried and resuspended in a lysis buffer composed of 7 *M* urea, 1% (*v/v*) protease inhibitor cocktail, and 2 m*M* EDTA (all reagents were purchased from Solarbio Biotech, Beijing, China, except for the protease inhibitor cocktail, which was purchased by MERCK, Darmstadt, Germany). After centrifugation at 25,000× *g* and 4 °C for 20 min, the supernatants were transferred into new tubes. Proteins in the supernatants were reduced with 10 mM dithiothreitol (Thermo Scientific, San Jose, CA, USA) at 56 °C for 1 h, alkylated with 55 mM iodoacetamide (Thermo Scientific, San Jose, CA, USA) for 45 min in the dark, and precipitated with six times their volume of precooled acetone (Sinopharm, Shanghai, China) at −20 °C for 2 h. The protein pellets were then washed twice with ice-cold acetone. The pellet was dissolved with the lysis buffer. The Bradford method [23] was conducted to measure the protein concentration of the solution with a 2-D Quant kit (Amersham BioSciences Corp, Marlborough, MA, USA).

In order to generate peptides, protein pellets were digested overnight with trypsin (Solarbio, Beijing, China) with a trypsin:protein mass ratio of 1:50, followed by a second digestion for 4 h with a trypsin:protein mass ratio of 1:100. The resulting peptides were desalted using a Strata X C18 SPE column (Phenomenex, Los Angeles, CA, USA) according to the manufacturer’s instructions. Peptides were then dissolved in 0.5 *M* tetraethylammonium bromide (Sigma-Aldrich, Saint Louis, MO, USA). Finally, the obtained peptides were labeled with the TMT kit (TMT 10 plex™ Isobaric Label Reagent Set, Thermo Scientific, San Jose, CA, USA).

#### 2.5.2. HPLC Fractionation and LC-MS/MS Analysis

The labeled peptides were fractionated on a Gemini C18 Column (5 μm, 250 × 4.6 mm column; Phenomenex, Torrance, CA, USA) using 5% acetonitrile (solvent A; U.S. Pharmacopeia, Rockville, MD, USA) and 95% acetonitrile (solvent B), and then fractionated on a SHIMADZU-LC-20AB system monitored at 214 nm. The fractions were collected using 1–5% solvent B for 10 min, 5–35% solvent B for 40 min, 35–95% solvent B for 1 min, solvent B for 3 min and then 5% solvent B for 10 min at a flow rate of 1 mL/min. A total of 20 fractions were obtained and vacuum dried for further LC-MS/MS analysis.

Before loading, each sample was dissolved in mobile phase A (2% acetonitrile, 0.1% formic acid; *v*/*v*). The peptide mixtures were separated in a SHIMADZU-LC-20AB system (SHIMADZU Corporation, Saitama, Japan) with an Acclaim Pep Map RSLC C18 analytical column (75 μm × 25 cm, 2 μm particle size; Thermo Scientific, Waltham, MA, USA) at a flow rate of 300 nL/min. The liquid gradient was set as followed: 8 min of 5% mobile phase B (0.1% formic acid, 98% acetonitrile), 35 min of 8–35% B, 5 min of 35–60% mobile phase B, 2 min of 60–80% mobile phase B, 5 min of 80% mobile phase B, and 10 min of 5% mobile phase B. The peptides separated by the SHIMADZU-LC-20AB system were injected into a Q Exactive (Thermo Fisher Scientific, Waltham, MA, USA). The peptides were ionized at 1.6 kV and data-dependent acquisition was conducted by Q Exactive. The MS1 spectrum was set at 350–1600 m/z, and the scanning resolution was set at 70,000 dpi. The MS2 spectrum was set at 100 m/z, and the scanning resolution was set at 17,500 dpi. Dynamic exclusion was set at 15 s. The automatic gain control setting of MS1 and MS2 were set at 3E6 and 1E5, respectively. The twenty most intense ions were selected for fragmentation by entering a higher-energy collision dissociation pool. Subsequently, secondary mass spectrometry analysis was performed. The TMT-proteomic analysis was conducted by BGI-Shenzhen, Shenzhen, China.

#### 2.5.3. Data Processing and TMT Quantification

Secondary mass spectrometry data were processed using *Mascot Search Engine* v 2.3.02 (Matrix Science, London, UK) and *Proteome Discoverer Software* v 2.1.0.81 (Thermo Scientific, USA) against *Bos taurus* sequences in Uniprot KB (*Bos taurus* database, 31,889 entries, downloaded 14 September 2019, http://www.uniprot.org/). Mascot and Proteome Discoverer were searched with a fragment ion mass tolerance of 0.050 Da and a parent ion tolerance of 20.0 ppm. IQuant (The Beijing Genomics Institute, Shenzhen, China, OR) was used to validate MS/MS-based peptide and protein identifications (Wen et al., 2014). The false discovery rate (FDR) thresholds for protein, peptide, and modification sites were set at 1%. For protein quantification, unique peptides with a report ion intensity of >10,000 and FDR of <0.01 were used, and the signal between different samples was normalized by the sum of the total intensity of each reported ion channel. Total protein abundances were normalized to one million by taking the median of all quantified proteins in a sample.

#### 2.5.4. Protein and Gene Ontology Identification

We used the Mascot Search Engine 2.3.02 (Matrix Science, London, UK) classification system to identify significantly enriched Gene Ontology (GO) terms and pathways.

#### 2.5.5. Identification of Proteins Related to the Phenotype of Interest

A partial least square regression (PLSR) analysis with sparsity-inducing penalized regression was fitted. The PLSR was performed by using the *R* package *caret* v 6.0 [24], with the formula set as train(NEFA + MDA + GPx + triacylglycerol ~., method = ‘pls’). Specific engines for variable importance on a model-by-model basis were performed by using the vamp() function in *caret* v 6.0 [24].

#### 2.5.6. KEGG Pathway Enrichment

The set of the top 20 variable importance proteins was enriched using KABOS 3.0 [25]. The background gene list was set as *Bos taurus*, the pathway database was set to the KEGG pathway, and a hypergeometric test/Fisher’s exact test was conducted. All defined KEGG pathways and their correlations were presented in a landscape, and the clusters of KEGG pathways were produced online (http://kobas.cbi.pku.edu.cn/, 22 September 2021) using the default settings.

### 2.6. Statistical Analyses

A pairwise *t*-test was performed for the phenotypic data by using *t.test()* in *R v 4.*0 [26], with the parameter of *paired = TRUE*. A *p*-value of ≤0.05 was set as the criterion for significance, and 0.05 < *p* ≤ 0.1 was defined as a tendency.

## 3. Results

**Figure 1:**
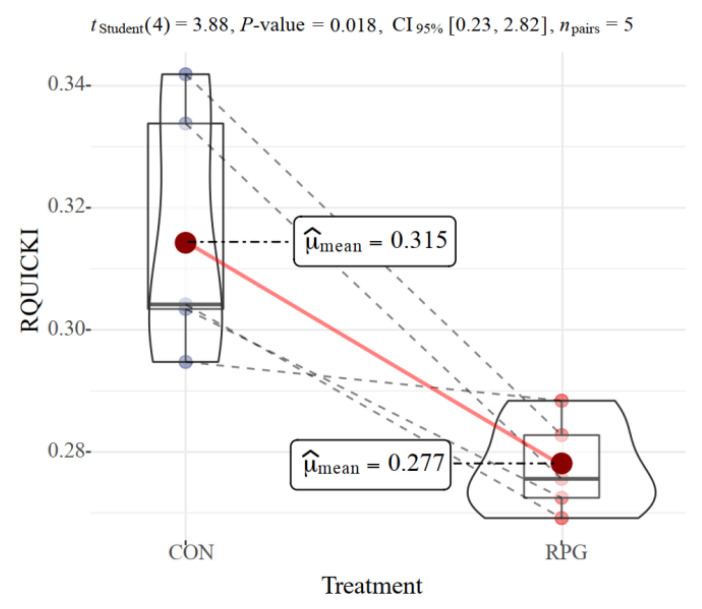
Effects of rumen-protected glucose (RPG) on RQUICKI, an insulin resistance index estimate according to the concentrations of NEFA, glucose, and insulin in plasma. Paired cows are connected with dashed lines.

The concentration of MDA was higher in the livers of RPG cows when compared to CON cows (*p* = 0.02; Table 1). The GPx activity was increased (*p* = 0.03) and the activities of CAT and SOD had a tendency to increase (*p* ≤ 0.07) in the livers of RPG supplemented cows. TAC was not influenced by RPG supplementation (*p* = 0.35), but triacylglycerol content in the liver was increased by RPG supplementation (*p* = 0.02). The RQUICKI insulin resistance index was decreased by RPG supplementation (*p* = 0.018, Figure 1).

**Figure 2:**
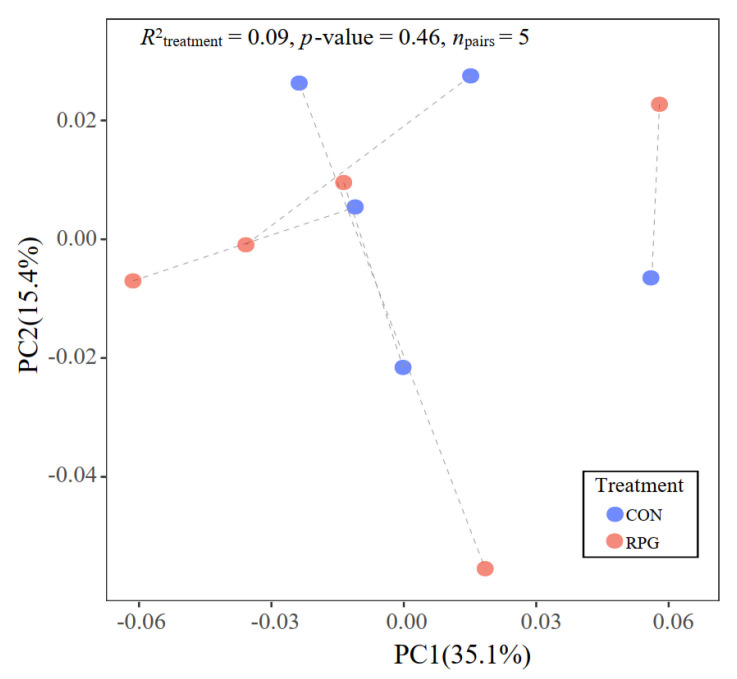
Principal coordinate analysis based on the Bray–Curtis dissimilarity matrix of liver protein profiles. Paired cows are connected with dashed lines.

A total of 4890 proteins were identified by GO in the liver samples (Supplemental Tables S2 and S3). Of these, most proteins were involved in synthesis of cellular components (3991/4890), molecular functions (3829/4890), and biological processes (3787/4890) (Figure S1). Principal coordinate plots did not cluster by treatment (*p* = 0.46, Figure 2).

**Figure 3:**
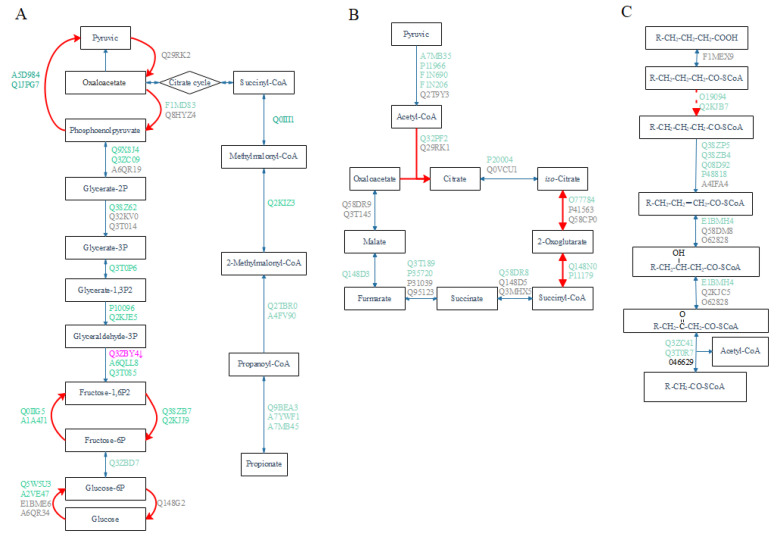
Hepatic metabolism pathway of glucose, citric acid cycle and fatty acid metabolism as influenced by rumen-protected protein (RPG) supplementation. (**A**) Glycolysis/glycogenesis. (**B**) Citrate cycle. (**C**) β-oxidation of fatty acid. The red arrows show the rate-limiting steps in each metabolic pathway. For each protein code, the gray color indicates no detection, green indicates no effect caused by RPG supplementation, and purple indicates a decrease in the RPG treatment group.

We filtered 35, 24, and 17 bovine proteins from the KEGG pathways of *Bos tarus* [27], and 71.4%, 58.3%, and 58.8% of them were identified by proteomics to be involved in glycolysis/glycogenesis, citric acid cycle, and fatty acid β oxidation, respectively (Figure 3). Most of the detected proteins were uninfluenced by RPG supplementation (*p* ≥ 0.15, Figure 3, Table S3), except for Q3ZBY4, involved in the production of fructose 1, 6-diphosphate from glyceraldehyde 3-phosphate, which was down-regulated by RPG supplementation (*p* = 0.03).

**Figure 4:**
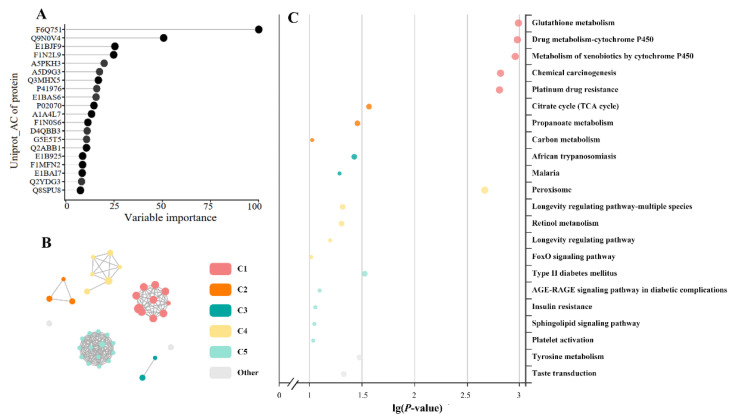
Liver protein profiles. (**A**) Top 20 most important proteins as found by a partial least squares regression associated with oxidative stress, fatty acid metabolism, and the concentration of NEFA in plasma, and of MDA, GPx, and triacylglycerol in the liver. (**B**) Network of enriched KEGG pathways. The nodes represent KEGG pathways, and the node colors represent different clusters; the node size represents 6 levels of enriched *p*-value (from smallest to largest): [0.05, 1], [0.01, 0.05], [0.001, 0.0), [0.0001, 0.00), [10^−10^, 0.0001), (0, 10^−10^]; and correlated pathways are linked with lines. The pathways in each of the clusters are listed in Table S5. (**C**) Bubbles of KEGG pathways enriched in the RPG treatment. The color and size of the bubbles are the same as the color and size in the circular network. If there are more than 5 enriched KEGG pathways per cluster, the top 5 with the highest enrichment ratio are displayed.

The top twenty proteins most associated with oxidative stress, fatty acid metabolism, and concentration of NEFA in plasma, and of MDA, GPx, and triacylglycerol in the liver, were F6Q751, Q9NOV4, E1BJF9, F1N2L9, A5PKH3, A5D9G3, Q3MHX5, P41976, E1BAS6, P02070, A1A4L7, F1N0S6, D4QBB3, G5E5T5, Q2ABB1, E1B92, F1MFN2, E1BAI7, Q2YDG3, and Q8SPU8 (Figure 4A and Supplemental Table S4). These proteins, identified through partial least squares regression, were involved in 37 KEGG pathways (Supplemental Table S5), which were grouped into six clusters (C1-C6; Figure 4B and Supplemental Table S5). Eighteen KEGG pathways were identified as enriched (Figure 4C, *p* < 0.05): glutathione metabolism, drug metabolism by cytochrome P450, metabolism of xenobiotics by cytochrome P450, chemical carcinogenesis, and platinum drug resistance in C1; citrate cycle and propanoate metabolism in C2; African trypanosomiasis pathway in C3; peroxisome in C4; type II diabetes mellitus in C5; and tyrosine metabolism in C6.

## 4. Discussion

Milk yield of our cows was considerably lower than the average for developed countries, but likely representative of the developing world [28]. This may explain why some of our results were inconsistent with those obtained with high-yielding cows. We observed increased concentration of circulating insulin and glucose [1], while Sauls-Hiesterman et al. [29] reported that no changes was caused by an even higher level of RPG supplementation (1500 g/d). Loncke et al. [30] reported a mean liver outflow of glucose of 0.995 (0.467–1.405) mmol kg BM^−1^ h^−1^, which for 515 kg cows (closer with the cows in our study), is equivalent to 2214 (1039–3126) g/d. In that regard, the 90 g/d RPG supplementation in our experiment would represent only 4.1% of the average gluconeogenesis flow, i.e., rather small. However, the lactation animals in the meta-analysis by Loncke et al. [30] were consuming on average 26.4 g DM/kg BM, which would be equivalent to 13.6 kg DM/d. Because cows in this study consumed approximately 8 kg DM/d, if their glucose outflow was proportional to their DMI (and without considering differences in diet composition), their expected liver glucose outflow would have been 1302 instead of 2214 g/d. Then, after correcting for this, RPG supplementation in the present study would represent about 6.9% of gluconeogenesis; that may partially explain why low-producing cows had a milk production response to a relatively small amount of RPG supplementation.

Milk production increases rapidly after calving, and as a consequence, the mammary gland has high demands for energy and glucose [31]. Although RPG supplementation increased the circulating glucose concentration [1], proteomic analysis showed little change in the liver glucose metabolism, with one of the enzymes involved in gluconeogenesis even experiencing a decrease in relative abundance. The concentration of circulating insulin drops significantly after calving, allowing the mammary gland, rather than other peripheral tissues, to preferentially take up and utilize glucose [32]. Meanwhile, glucose utilization by peripheral tissues other than the mammary gland is reduced due to reduced insulin sensitivity [33]. The most accurate way to measure the insulin resistance is through hyperinsulinemic-euglycemic clamp studies [34]. However, the hyperinsulinemic-euglycemic clamp test is time-consuming and may stress the postpartum cows. Some simple and cheap surrogate indices have been developed in human medicine to assess insulin sensitivity in patients with diabetes. We conducted a simple test called the RQUICKI index [18], which is based on a single blood sample, to predict the insulin resistance of cows. Rumen-protected glucose was originally designed to relieve the negative energy balance betweem energy and glucose. We observed that RPG supplementation further reduced insulin sensitivity in cows, which partly explains the increased lipid mobilization, as reflected by a higher amount of circulating NEFA, caused by RPG. Most [35,36,37], although, not all [9], previous studies showed the result that cows had lower insulin sensitivity when infused with glucose in blood, in agreement with this study.

Fat mobilization in the post-peripartum period is a consequence of a negative energy balance and insulin resistance [38]. The preferential supply of milk to the offspring increases the burden on the maternal energy metabolism. The release of NEFA to the blood may exceed the oxidative capacity of the liver [39]. Under these conditions, triglycerides can accumulate in the liver, causing the metabolic disease known as fatty liver disease [40]. In this study, RPG supplementation increased triglyceride concentration in the liver, indicating that supplementation with RPG increased the risk of disturbances in the hepatic metabolism in early postpartum dairy cows. In contrast, a previous study with early postpartum ewes showed that intravenous glucose infusion reduced the mobilization of lipid reserves [41]. However, the energy deficiency in early postpartum ewes may not be as severe as in dairy cows, as indicated by similar plasma glycerol and milk yield between ewes infused with glucose and their control counterparts [41]. The lack of changes observed in the liver proteins suggest that adipose tissue can increase the liver’s ability to mobilize fat, but it has no ability to oxidize it. Other lipid marker in liver cells such as Annexin V/PI and Oil Red Staining could be useful to confirm lipid accumulation [42].

A high level of lipolysis in body adipose tissue may lead to increased oxidative stress in the liver. ROS-induced oxidative damage to lipids causes MDA accumulation, which is an index of lipid peroxidation. Oxidative stress caused by excess lipolysis is common in postpartum cows [40]. In this study, although ROS were not determined, the cause of the increased MDA with RPG supplementation could be explained by an increased oxidative stress. In the early postpartum dairy cow, the glucose absorbed in the intestinal tract is mainly used to synthesize lactose and glucogenic amino acids in milk, increasing the burden on the liver to oxidize fatty acids. Hepatic oxidative stress induced by glucose infusion has been reported in rats [43,44], but we are not aware of studies investigating the effects of glucose infusion on oxidative stress and antioxidant status in the liver of early postpartum cows. Mammals are equipped with an integrated antioxidant system that removes the harmful effects of ROS [45]. Hepatic oxidative stress state is not always reflected by the antioxidant markers in blood, as indicated by the inconsistency of superoxide dismutase activity between plasma and liver [46]. This inconsistency in superoxide dismutase activity further emphasizes the need to use liver tissue to study the antioxidant capacity. The superoxide is a primary ROS, and SOD is considered to be the first enzymatic antioxidant converting the superoxide to hydrogen peroxide [47]. Hydrogen peroxide is further degraded to water by CAT and GPx [45]. In this study, both SOD and CAT tended to increase and GPx increased in RPG supplemented cows, which indicates that RPG supplementation increased the oxidative stress and antioxidant activity. Oxidative stress is a consequence of an increased generation of ROS and/or the reduced physiological activity of antioxidants. Supplementation with RPG led to an increase of both oxidative stress and the activities of antioxidant enzymes, suggesting that RPG did not negatively affect the liver’s antioxidant response and that the aggravation of oxidative stress in the liver mainly resulted from the production of ROS exceeding the functional capacity of antioxidants. Previous evidence showed that increased oxidative stress exacerbates insulin resistance by impairing the secretion of insulin by β-cells [48,49]; however, RPG supplementation to the same cows used in this study elicited a positive response in circulating insulin concentration before feeding [1]. In a previous study, supplementation with RPG did not decrease circulating insulin concentration in early lactation dairy cows, in agreement with our results [9]. Increased insulin concentration suggests that the oxidative stress induced by RPG supplementation was not severe enough as to affect β-cell function.

The analysis of proteomic profiles in tissues has been proposed as an approach to identify metabolic alterations for medical and nutritional treatments, and markers of clinical status [15,16]. The amount of protein we identified was comparable to a previous report with early postpartum Holstein dairy cows [50]. As far as we know, this is the first study reporting the effects of exogenous glucose on liver protein profiles in dairy cows. The TMT-based untargeted proteomics analysis could not classify animals based on the proteomics alteration induced by RPG supplementation. Likewise, PCoA plots did not separate by treatment, indicating that major proteins in the liver were not affected by RPG supplementation. A minor proportion (68/2741) of proteins in the livers of cows suffering from a negative energy balance were reported to be different from those of cows not suffering from a negative energy balance [51]. Proteins differing between the cows with a negative energy balance and cows not undergoing a negative energy balance were involved in inflammatory response, mitochondrial dysfunction, and fatty acid uptake [51]. Replenishing energy with RPG was expected to shift hepatic protein profiles to be more similar to cows not suffering negative energy balance. Based on the hepatic metabolism induced by RPG, we selected a group of proteins that may contribute to increased lipolysis, ROS, and antioxidants by RPG supplementation. A protein may participate in more than one metabolic pathway; therefore, multiple KEGG metabolic pathways were found to be enriched [25]. These pathways were grouped into six major clusters based on the network features of the proteins involved. The glutamine pathway associated with antioxidant stress appeared in the C1 metabolic pathway cluster, and the most notable proteins associated with our response variables were F6Q751 (Glutathione transferase) and Q9N0V4 (Glutathione S-transferase Mu 1), which are members of the glutathione transferase (GST) superfamily. The GST family represents one of the most abundant and important series of detoxification enzymes in the liver [52]. The main function of GSTs is to conjugate electrophilic compounds with glutathione (GSH), thereby making these compounds less active and enabling their excretion [53]. In this way, GSTs contribute to the metabolism of drugs, pesticides, and other xenobiotics [54]. GPx reduces hydrogen peroxide and organic hydroperoxides to mitigate oxidative stress; therefore, the enriched metabolic pathway of glutamine could help prevent deleterious cellular events that might develop in the livers of early postpartum dairy cows supplemented with RPG. The citrate cycle pathway, associated with antioxidant stress, appeared in the C2 metabolic pathway cluster, and the most notable protein was succinate-CoA ligase. According to the characteristics of substrate formation, succinate-CoA ligase can be divided into two categories: ATP and GTP biosynthesis [55]. The former is mainly found in the brain and heart, while the latter is mainly found in the liver and kidneys [55]. Succinate-CoA ligase catalyzes the hydrolysis of succinyl-CoA thioester bond to produce GTP and succinate, which enters the citrate cycle. Inhibition of succinate-CoA ligase in rat liver mitochondria was shown to cause metabolic disorders such as severe lactic acidosis and fatty liver disease [55]. The peroxisome and type II diabetes mellitus pathways were selected in pathway clusters C4 and C5, respectively. The main protein that caused peroxisome pathway enrichment was P41976, or Mn-superoxide dismutase. TSODs are enzymes that catalytically convert superoxide radicals to oxygen and hydrogen peroxide. The active center of SODs binds to a metal atom, which can be Cu, Fe, Mn, or Ni [56]. Mn-SODs are well conserved throughout evolution and across kingdoms. The highest contents of Mn-SOD are found in the liver, followed by the kidney and heart [46]. Under severe oxidative stress such as aging [57] or high intake of alcohol [58], the expression of Mn-SOD in the liver increases dramatically, indicating its importance in resisting oxidative stress. A0JNH7, or protein kinase C, was the main protein enriched in the type 2 diabetes pathway. The protein kinase C family is composed of lipid-dependent kinases with wide-ranging roles in modulating insulin function [59]. Increased protein kinase C activity is linked to reduced insulin receptor autophosphorylation in the livers of starved rats [60]. Thus, an increase in its abundance with RPG supplementation suggests that the livers of RPG cows are in a state of energy deficiency.

## 5. Conclusions

Our study showed that RPG supplementation reduced insulin sensitivity and increased the triacylglycerol contents and oxidative stress in the livers of early postpartum cows. Proteins related to oxidative stress, lipolysis, and antioxidant function that were differentially expressed between the control and RPG treatments were classified as being involved in metabolic pathways of the glutamine pathway, citrate cycle, peroxisome, and type II diabetes mellitus, which could indicate an increased risk of metabolic disorders in the livers of early postpartum dairy cows supplemented with RPG. In this study, the risk of metabolic disorders seemed to be augmented by RPG supplementation to relatively low-yielding cows; further research with high-yielding dairy cows, which are more exposed to liver metabolic disorders, is recommended.

## Figures and Tables

**Table 1:** Effects of rumen-protected glucose (RPG) on the variables of oxidative stress, antioxidant capacity, and triglyceride content in the liver of early postpartum cows (*n*_pairs_ = 5).

Item ^1^	Treatment ^2^		SEM	*p*-Value
	CON	RPG		
Oxidative stress				
MDA, μmol/mg protein	0.68	1.09	0.116	0.02
Antioxidant				
TAC, μmol/mg protein	243	217	24.2	0.35
SOD, U/mg protein	1.24	2.02	0.315	0.07
CAT, U/mg protein	25.1	25.7	0.18	0.06
GPx, U/mg protein	27.6	52	8.04	0.03
Triacylglycerol, μmol/g	540	958	115.8	0.02

^1^ MDA—Malondialdehyde; TAC—total antioxidant capacity; SOD—Superoxide dismutase; CAT—Catalase; GPx—Glutathione peroxidase. ^2^ CON—cows supplemented with coating fat; RPG—cows supplemented with rumen-protected glucose.

## Data Availability

The proteomic data is contained within the article For more details, please check https://ngdc.cncb.ac.cn/omix: accession no. OMIX916; 9 February 2022.

## Supplementary Materials

The following supporting information can be downloaded at: https://www.mdpi.com/article/10.3390/antiox11030469/s1, Table S1. Ingredients and chemical composition of basal diet; Table S2. Protein annotation; Table S3. Protein profiles; Table S4. Variable importance. Table S5. Pathway enrichment analysis; Figure S1: Gene Ontology (GO) terms of total proteins for categories of cellular component.
