# Supplementary material for: Supplementation of Rumen-Protected Glucose Increased the Risk of Disturbance of Hepatic Metabolism in Early Postpartum Holstein Cows
Source: Antioxidants (Basel). 2022 Feb 26;11(3):469. doi: 10.3390/antiox11030469 (PMC8944473; doi:10.3390/antiox11030469)
Supplement: Supplementary file 1 [file antioxidants-11-00469-s001.zip › Jan26 Supplemental TableS1 and FigureS1.pdf]

Table S1. Ingredients and chemical composition of basal diet.

Item	
Ingredient, g/kg	
Corn silage	302.0
Wheat bran	213.0
Oat hay	128.0
Alfalfa hay	171.0
Soybean meal (49% CP)	81.0
Ground shelled corn	70.0
Calcium carbonate	11.3
Salt	4.5
Sodium bicarbonate	6.8
Magnesium oxide	0.9
Potassium chloride	3.2
Vitamin and mineral premix[1]	4.1
Chemical composition (g/kg DM)	
CP	146.0
EE	21.0
Starch	148.0
NDF	452.0
ADF	255.0
Ash	60.0
Calcium	9.8
Phosphorus	5.4
NE_L[2], MJ/kg DM	5.73

[1] Premix contained (per kg) 3040 mg of Cu; 3170 mg of Fe; 14, 280 mg of Zn; 3060 mg of Mn; 40 mg of Co; 180 mg of I; 100 mg of Se; 1250 KIU of vitamin A; 270 K IU of vitamin D3; and 5000 IU of vitamin E.

[2] NE_L was estimated according to NRC (2001).

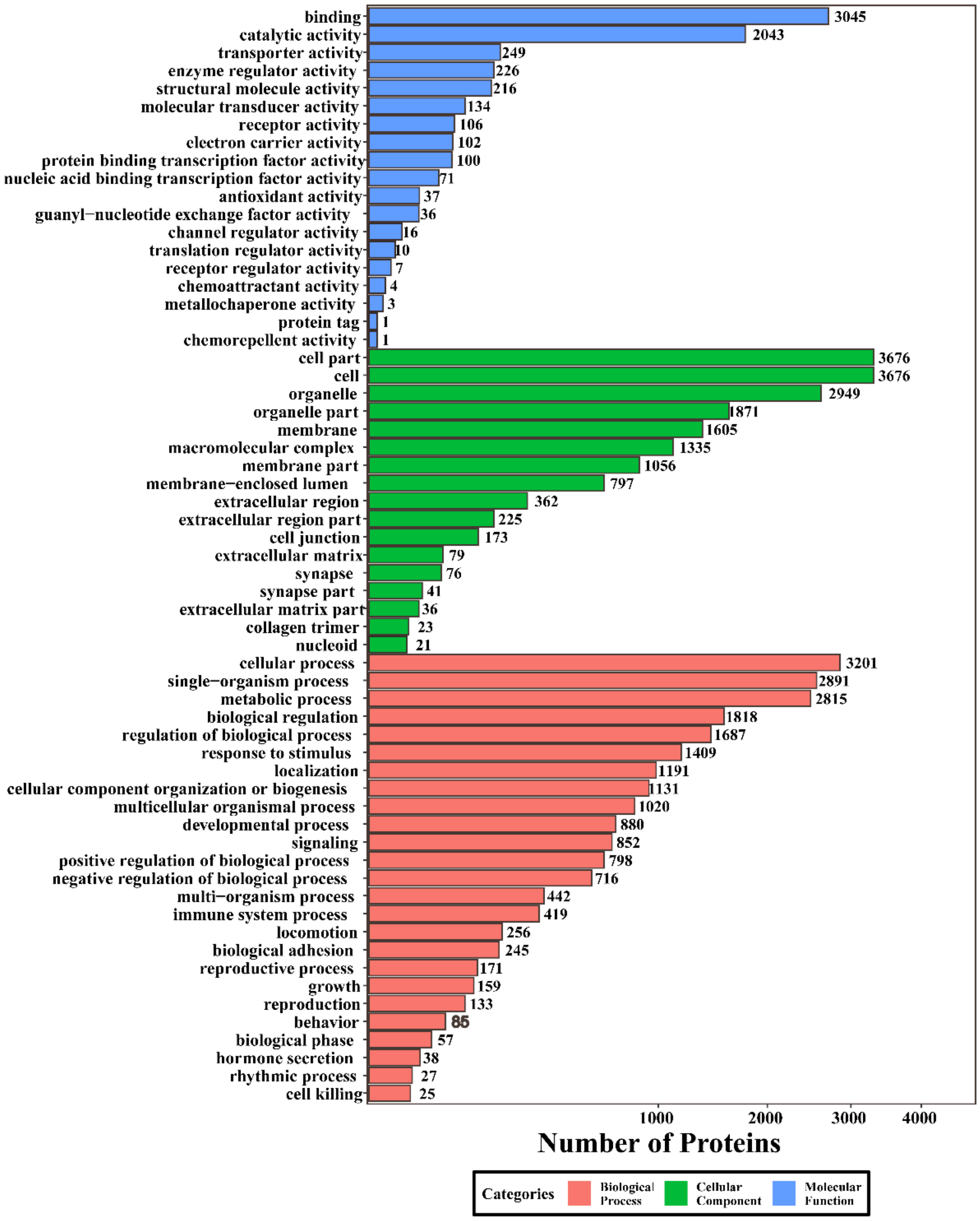

Figure S1. Gene Ontology (GO) terms of total proteins (based on java TreeView) for categories of cellular component, in green; molecular function, in blue; and biological process, in red for proteins in liver tissue of early lactation cows. The x-axis shows the number of the proteins, and the y-axis indicates the information about GO annotation.
